# The Korea National Disability Registration System

**DOI:** 10.4178/epih.e2023053

**Published:** 2023-05-11

**Authors:** Miso Kim, Wonyoung Jung, So Young Kim, Jong Hyock Park, Dong Wook Shin

**Affiliations:** 1 Department of Family Medicine/Supportive Care Center, Samsung Medical Center, Sungkyunkwan University School of Medicine, Seoul, Korea; 2 Department of Public Health and Preventive Medicine, Chungbuk National University Hospital, Cheongju, Korea; 3 College of Medicine/Graduate School of Health Science Business Convergence, Chungbuk National University, Cheongju, Korea; 4 Department of Preventive Medicine, Chungbuk National University College of Medicine, Cheongju, Korea; 5 Department of Clinical Research Design & Evaluation, Samsung Advanced Institute for Health Science & Technology (SAIHST), Sungkyunkwan University, Seoul, Korea

**Keywords:** Disability, Database, Epidemiology

## Abstract

The Korea National Disability Registration System (KNDRS) was established in 1989 to provide social welfare benefits based on predefined criteria for disability registration and an objective medical assessment using a disability grading system. Disability registration requires (1) a medical examination by a qualified specialist physician and (2) a medical advisory meeting to review the degree of disability. Medical institutions and specialists for the diagnosis of disabilities are legally stipulated, and medical records for a specified period are required to support the diagnosis. The number of disability types has gradually expanded, and 15 disability types have been legally defined. As of 2021, 2.645 million people were registered as disabled, accounting for approximately 5.1% of the total population. Among the 15 disability types, disabilities of the extremities account for the largest proportion (45.1%). Previous studies have investigated the epidemiology of disabilities using data from the KNDRS, combined predominantly with data from the National Health Insurance Research Database (NHIRD). Korea has a mandatory public health insurance system that covers the entire Korean population, and the National Health Insurance Services manages all eligibility information, including disability types and severity ratings. In short, the KNDRS-NHIRD is a significant data resource for research on the epidemiology of disabilities.

## GRAPHICAL ABSTRACT

[Fig f4-epih-45-e2023053]

## INTRODUCTION

The World Health Organization (WHO) published the International Classification of Impairments, Disabilities and Handicaps (ICIDH) in 1980 and established a conceptual framework for disabilities. This classification system involves 3 categories: impairment, disability, and handicap [1]. The system was revised and renamed in 2001 to the International Classification of Functioning, Disability, and Health (commonly known as ICF). The ICF combines the biological and social aspects of disability. From this perspective, “disability” is an umbrella term for impairment, activity limitation, and participation restriction, the latter of which refers to negative interactions between an individual and situational factors [2].

Although the direction of disability policy has been agreed upon internationally, different countries have different models of disability assessment, and no model covers all aspects of disability. The conceptual classification of disability proposed by Bernell [3] consists of 3 facets: medical, functional limitation, and socio-political. The classification system adopted by European countries can be broadly divided into 3 categories: procedural, capacity profile, and impairment [4]. Among these approaches, the disability assessment method differs depending on the approach adopted by a specific country.

Korea’s disability registration system has been regularly revised to keep pace with international standards. In 1981, the country enacted the Welfare Act for the Mentally and Physically Disabled. Then, in 1989, it established a national registration system for people with disabilities. After undergoing several revisions by 2019, the Act on Welfare of Persons with Disabilities was established, which defines a disability as a physical or mental condition that significantly limits an individual’s daily life or social life for the long term. Although the Korea National Disability Registration System (KNDRS) has shifted from medically focused solutions to more interactive ones, it continues to focus on a medical or impairment approach and has used objective measures to assess disability. Because Korea has a unique identification number system, KNDRS data can be linked to national health data. Currently, registration information (disability type and severity) is provided as part of the Korean National Health Information database. However, there is no English-language reference article for consultation by related academic societies.

In this article, we (1) explain the KNDRS, including its history and the disability registration procedures, (2) examine the 15 disability types, and (3) outline current statistics of Koreans with disabilities as of 2019 and introduce studies using KNDRS data.

## KOREA NATIONAL DISABILITY REGISTRATION SYSTEM

### History

Korea’s welfare policy for people with a disability was systematically implemented at the national level when the Welfare Act for the Mentally and Physically Disabled was enacted in 1981. It was amended in 1989 and is now known as the Act on Welfare of Persons with Disabilities. The previous law was more declarative rather than reflecting the needs of the disabled, while the current law has been amended to ensure the human rights and independence of people with disabilities. The KNDRS was introduced in 1989 and has been used as a criterion for prioritizing welfare projects for the disabled and for selecting subjects by judging the disability type and severity.

The type and severity of disability are legally defined in Korea. Until recently, these definitions have been medically-based. The number of legally defined disability types has been gradually expanded, increasing from 5 to 10 in 2000 and from 10 to 15 in 2003 (Supplementary Material 1). Additionally, in 2007, the term “developmental disability” was renamed “disability due to autism,” and “mental retardation” was renamed “intellectual disability.” Therefore, the current 15 disability types include disability of the extremities, vision, hearing, and speech and language and disabilities due to brain injury, facial deformity, renal failure, heart problems, liver disease, respiratory problems, ostomy, epilepsy, intellectual disorders, autism, and mental disorders. These categories are broadly divided into physical disabilities and mental disabilities. Physical disabilities are further divided into impairment of external and internal body function, and mental disabilities are divided into developmental impairment and psychiatric impairment (Table 1).

Each type of disability is graded according to its severity. This disability grading system was introduced in 1989. It was set based on the disability rating, with grade 1 being the most severe and grade 6 being the mildest. The disability rating was determined as a percentage reflecting the ratio between the loss of function due to impairment and function before loss. Grades 1-6 corresponded to ratios greater than 85%, 75-84%, 60-74%, 45-59%, 35-44%, and 25-34%, respectively.

In 2019, the disability grading system was abolished in response to the opinion that services for people with a disability should be uniformly provided according to a medical assessment. Therefore, to provide personalized services, in addition to considering medical factors, the system has been changed to reflect social and environmental factors and the desires of individuals with disabilities and to comprehensively evaluate a person’s basic work ability.

First, the degree of disability is classified as severe or mild. The existing grades 1-3 are recognized as severe disabilities, while grades 4-6 are recognized as mild.

Second, a comprehensive assessment tool is being developed that reflects not only the severity of physical disability, but also socioeconomic conditions and activity levels [5] to evaluate the degree of disability. Socioeconomic conditions include property, income, residential area, family relationships, and so forth. Activity levels are assessed based on the activities of daily living (ADL) and instrumental activities of daily living (IADL). Therefore, welfare services can be provided with consideration of socioeconomic factors and ADL rather than simply evaluating the degree of disability based on a medical assessment.

In summary, the KNDRS prior to 2019 has the advantage of being objective and suitable for research because it is based on medical assessments, but it has limitations that it does not reflect other aspects of disability, such as socioeconomic status and functional status. To overcome this limitation, the activity level is evaluated in terms of ADL and IADL as a functional status assessment, and the socioeconomic status of disabled people is also considered.

### Disability assessment and registration

As mentioned above, the concept of disability in Korea’s disability assessment method is still based on a medical approach or impairment assessment. Disability registration requires several legal procedures. First, a person who intends to register as a person with disability should receive a medical examination and disability certificate from a qualified specialist physician in the relevant field (Table 2) and submit it to the local community center with other required documents. That is, for each disability, a specialist in the relevant field evaluates the disability at a qualified medical institution. For example, in the case of hearing disability, an otolaryngologist in a hospital with an audiometric booth evaluates hearing impairment. Second, the National Pension Service Office holds a medical advisory meeting composed of 2 or more specialist doctors, reviews the degree of disability based on the submitted documents, and provides the result to the local community center. At that point, a disability registration card can be issued.

**Figure 1.**
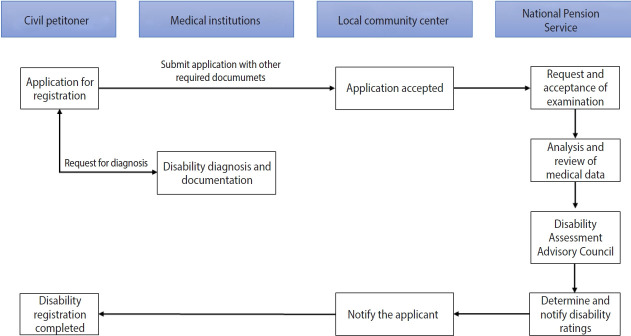
Disability registration application process in Korea.

Figure 1. Medical institutions and specialists for the diagnosis of disabilities are legally stipulated. For each type of disability, medical records for a specified period are required to support the diagnosis. For example, a disability due to a heart problem requires medical records to prove that the condition lasted for at least 1 year, whereas 3 months is required for a disability due to renal failure, 2 months for a disability due to liver disease, and 6 months for an epilepsy disorder. Other details are given in Table 2.

### Types of disabilities in Korea

There are 15 legally defined types of disabilities in Korea. Their brief definitions and specific criteria are described in the following sections.

#### Disability of the extremities

Disability of the extremities includes (1) amputation (Supplementary Materials 2 and 3), (2) joint disorders (Supplementary Materials 4 and 5), (3) dysfunction of the extremities (Supplementary Materials 6 and 7), (4) spine disorders (Supplementary Material 8), and (5) other disorders such as deformities (Supplementary Material 9). The main diagnoses of the causes of extremities disability are spinal diseases, joint diseases, and fractures. In most cases, physical examinations and X-ray tests are required to diagnose disability of the extremities.

Grades are determined by disability severity. For example, subjects belonging to grade 1 in each category of disability of the extremities are as follows: (1) amputation: amputation above the wrist joint of both arms (upper extremities) or amputation of both legs above the knee joint (lower extremities); (2) joint disorders: the range of motion of the shoulder, elbow, and wrist joints of both arms is decreased by more than 75% (upper extremities) or the range of motion of the hip, knee, and ankle joints of both legs is decreased by more than 75% (lower extremities); and (3) dysfunction: complete paralysis of both arms or legs (manual muscle strength grades 0, 1). Spine disorders and other disorders such as deformities are not categorized as a grade 1 disability.

#### Disability due to brain injury

Disability due to brain injury is defined as a physical disability caused by organic lesions of the brain, such as cerebral palsy, traumatic brain injury, or stroke, causing significant restrictions in walking and daily activities. Disability severity is generally determined according to the modified Barthel Index, which measures physical disability relating to activities of daily living, mostly for stroke patients [6] (Supplementary Material 10). Subjects belonging to grade 1 of disability due to brain injury are (1) unable to walk independently and require total assistance from others; (2) unable to perform any ordinary activity owing to complete paralysis of both arms and require total assistance from others; and (3) unable to conduct any ordinary activity owing to complete paralysis of 1 arm and 1 leg and require total assistance from others. Furthermore, (4) the modified Barthel Index is less than or equal to 32 points, and the individual requires total assistance from others to perform all ordinary activities including ambulation (Supplementary Material 11).

#### Visual disability

Visual disability includes vision impairment and visual field defects. Disability due to loss or reduction of vision is divided into degrees. The state of complete absence of vision is called total blindness (0 vision), the state of recognition of flickering light rays in a dark room is called light perception, the ability to recognize when a hand moves left and right is expressed as hand movement, and the state of being able to count the number of fingers presented 1 m in front of the subject is expressed as finger count (visual acuity of 0.02 or less). When the eyes focus on a given point, the range in which the eyes can perceive is called the visual field. A disability can be identified even when the range of the visual field is narrow. For visual disability, grade 1 means that the visual acuity of the stronger eye is less than or equal to 20/1,000. Visual field defects are not categorized as a grade 1 disability, but begin at grade 3 (Supplementary Material 12). In total, 75.9% of people in this category had disabilities due to acquired causes, of which sensory organ diseases, accidents, and trauma accounted for large proportions.

#### Hearing disability

Hearing disability is divided into hearing impairment and balance disorder. Hearing impairment can only be diagnosed by an otolaryngologist at a medical institution with an audiometric booth. Examples of diseases that cause hearing disability are sensory organ diseases, unknown diseases, infectious diseases, and toxicity. In hearing disability, grade 2 means that the average hearing loss over 4 frequencies (0.5, 1.0, 2.0, and 4.0 kHz) in both ears is greater than 90 dB (Supplementary Material 13). Balance disorder refers to a disturbance in the equilibrium function of the body due to damage to the hearing function, resulting in challenges in daily life. According to the level of walking straight and performing daily activities, a grade from 3 to 5 is assigned (Supplementary Material 14).

#### Speech and language disability

Speech and language disability is defined as a disability rendering a person unable to adjust normally in social life owing to a disturbance in communication. It includes articulation disorder, voice disorder, fluency disorder, and other language disorders due to cerebral palsy, intellectual disability, and hearing disability. The most severe disability of this type is grade 3, where it is not possible for the person to produce sounds or conduct a simple conversation using an electrolarynx or esophageal voice (Supplementary Material 15).

#### Facial deformity disability

Facial deformity disability includes disability due to facial deformation, such as scarring, depression, and thickening. Facial deformity disability causes limitations in social activities owing to the uncomfortable gazes of others. Accidents accounted for 72.8% of the causes of facial deformity disability. Among cases of accident-caused disability, 52.6% are due to burns. Facial deformities are graded from 2 to 5 based on the degree of deformation (Supplementary Material 16).

#### Disability due to renal failure

Disability due to renal failure occurs in a person who must undergo hemodialysis or peritoneal dialysis due to renal dysfunction or who has significant limitations in daily life due to permanent impairment of renal function. Those who have regularly undergone hemodialysis or peritoneal dialysis for more than 3 months are classified as grade 2. Post-kidney transplantation status is assigned grade 5 (Supplementary Material 17). The most frequent causes of kidney failure are diseases of the urogenital or endocrine system [7].

#### Disability due to heart problems

Disability due to heart problems occurs in a person who has significant limitations in daily life due to disorders such as dyspnea caused by impairment of heart function. Disability due to heart problems is graded based on clinical findings and test results (details in Supplementary Material 18). Those who have persistently reduced heart function with angina syndrome at rest and have a sum of clinical findings and test results of 30 points or higher are classified as grade 1 (Supplementary Material 18).

#### Disability due to liver disease

Disability due to liver disease involves a significant restriction in daily life due to chronic liver failure and its complications. In Korea, the prevalence of liver cirrhosis is particularly high. Hepatitis B virus infection is the most common cause of liver cirrhosis, followed by alcohol use and hepatitis C virus [8]. Residual liver function classified as Child–Pugh C class due to chronic liver disease (e.g., liver cirrhosis or hepatocellular carcinoma) and observed hepatic encephalopathy or diuretic-refractory ascites are classified as a grade 1 disability. Post-liver transplantation status is grade 5 (Supplementary Material 19).

#### Disability due to respiratory problems

Disability due to respiratory problems takes place when a person experiences considerable restrictions in daily life due to chronic dysfunction of the respiratory organs such as lungs and bronchial tubes. The degree of dyspnea, pulmonary ventilation function (determined by a pulmonary function test), and arterial oxygen partial pressure (determined by arterial blood gas analysis) determine the degree of disability. Disability due to respiratory problems is categorized as grade 1 if oxygen therapy is required even at rest, the normal forced expiratory volume-one second is less than 25% of the predicted value, or the arterial oxygen saturation at rest in the air is less than 55 mmHg. Requiring a ventilator all day in the presence of a tracheostomy tube due to chronic respiratory disease is also considered a grade 1 disability. Lung transplantation status or a pleural fistula is grade 5 (Supplementary Material 20).

#### Disability due to ostomy

Disability due to ostomy is when a person has a stoma or urostomy due to a disorder of bowel or urination function and is significantly restricted in daily life. A stoma is an opening surgically produced in the colon through the abdomen. A urostomy is a surgical opening in the lower abdomen that allows urine to drain out of the body. There is no grade 1 disability in this category; it is classified as grade 2 if both a stoma and urostomy are present and accompanied by complications (Supplementary Material 21).

#### Disability due to epilepsy

Disability due to epilepsy is defined as severe restriction in daily or social life due to damage to brain nerve cells caused by epilepsy. Epilepsy is a disease in which convulsive seizures and loss of consciousness occur repeatedly. It is not a grade 1 disability; it begins at grade 2. The specific criteria differ between adults (Supplementary Material 22) and children (Supplementary Material 23). Details on the diagnosis of disability due to epilepsy have been described in a review article by Cho & Kim [9] in 2021.

#### Intellectual disability

Intellectual disability is determined according to the intelligence quotient (IQ) obtained by conducting personal intelligence tests, such as the Wechsler Intelligence Scale for Children, which is one of the most commonly used IQ tests. This test measures a child’s intellectual ability and the 5 cognitive domains that impact intellectual performance [10] and references the social maturity scale that measures an individual’s growth or change based on the Vineland Social Maturity Scale. For intellectual disability, grade 1 refers to an IQ less than 35 and marked difficulty in adapting to daily and social life without assistance of others (Supplementary Material 24).

#### Disability due to autism

Disability due to autism follows the diagnostic criteria of the 10th International Classification of Disease (ICD-10), the official classification system for mental disabilities in Korea. Grade 1 severity of disability due to autism includes pervasive developmental disorder (autism spectrum disorder) according to the ICD-10 code with an IQ less than 70 and a Global Assessment Scale (GAS) score less than 20 (Supplementary Material 25). The GAS is designed to measure the daily functioning of children with autism spectrum disorder.

#### Disability due to mental disorders

To be diagnosed with a disability due to a mental disorder, documents must prove that the patient has been treated regularly for 1 year immediately prior to the diagnosis of the disability. This disability includes schizophrenia, schizoaffective disorder, bipolar affective disorder, and recurrent depressive disorder, among others. Disability grades for mental disorders are determined in the following process. First, the current treatment status is assessed. Second, the diagnosis of a mental disorder and the date of the initial diagnosis are confirmed. Third, the impairment caused by the mental disorder is identified. Next, the status of the disability caused by the mental disorder is assessed. Finally, the mental disorder grade is comprehensively determined. A disability is evaluated based on 6 criteria: having the ability to (1) properly feed oneself; (2) keep oneself clean, such as toileting, bathing, and washing; (3) communicate appropriately and demonstrate cooperative interpersonal skills; (4) take medication at the correct dose and time without assistance; (5) manage financial matters; and (6) use transportation. In addition, the Global Assessment of Functioning is used to make a comprehensive determination of mental disorder grade. This scale is not a testing tool for diagnosis; rather, it is used to evaluate an individual’s current functional status regardless of the diagnosis. Its scores range from 1 point to 100 points [11] (Supplementary Material 26).

### Current disability statistics

**Figure 2.**
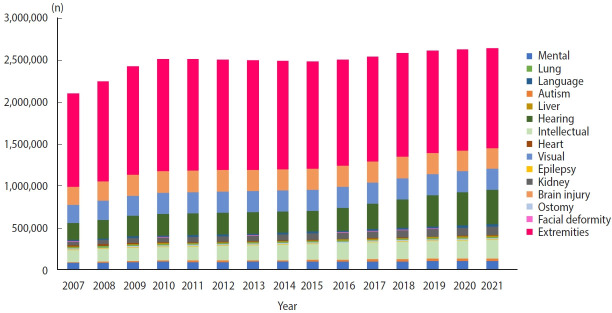
Number of registered people with disabilities by disability type and calendar year.

**Figure 3.**
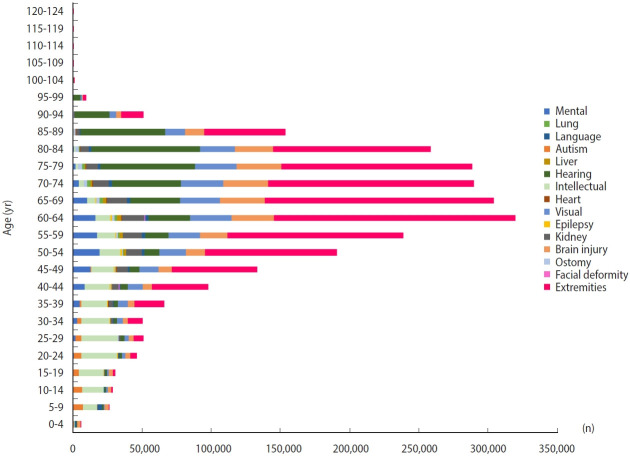
Number of registered people with disabilities by disability type and age in 2021.

As of 2021, 2.645 million people were registered as disabled, accounting for approximately 5.1% of the total population. Among the 15 types of disabilities, the proportion of disability of the extremities (45.1%) was the highest, but it has decreased over time. Meanwhile, disability of hearing, disability of intellect, disability due to autism, and disability due to renal failure have been becoming increasingly frequent (Figure 2) [12]. Males outnumbered females in all disability types, with males accounting for 57.7% of the total in 2021 (Table 3). The age groups of 60-79 and 70-79 had the highest numbers of registered disabled people. The proportion of intellectual disability was highest in those younger than 30, that of disability of the extremities varied among age groups ranging from the 40s to the 80s, and that of hearing disability was highest in those aged 90 years or more (Figure 3). The geographical distribution of disabilities in Korea in 2018 (reflecting the most recent data) is also presented in Supplementary Material 27. The proportions of disabilities did not vary substantially across the regions.

Various studies have used data from the Korea National Disability Registration Database. These data have often been combined with those of the NHIRD, which are provided by the National Health Insurance Service (NHIS) in Korea [13]. Korea has a mandatory public health insurance system that covers the entire Korean population, and the NHIS manages all eligibility information, including the disability type and severity level. In addition, there is a national health screening program that includes lifestyle assessment (smoking, alcohol consumption, exercise, etc.), body measurements (body mass index, waist circumference, blood pressure, etc.), and blood and urine tests. These data are also included in the NHIRD [14].

Some examples of research findings using this data are as follows: (1) disparities were identified in national cancer screening participation between people with and without disabilities (cervical cancer [15], gastric cancer [16], colorectal cancer [17], liver cancer [18], and breast cancer [19]), showing that the cancer screening rate varies depending on the type and severity of disability and suggesting significant disparities among people with disabilities; (2) differences were found in cancer treatment and prognosis; for instance, less intensive treatment resulted in a lower survival rate for multiple myeloma [20], prostate cancer [21], acute myeloid leukemia [22], lung cancer [23], and gastric cancer [24]; (3) higher risks of adverse health events were found in people with disabilities than in people without disabilities, including cardiovascular disease [25], fracture [26], tuberculosis [27], complicated appendicitis [28], obesity [29], and suicide [30].

To the best of our knowledge, the use of national-based or population-based systems to register disabilities based on predefined medical criteria is rare. In addition, linkage to healthcare claims data is an approach with unique prospects in Korea. In summary, the KNDRS-NHIRD is expected to serve as a significant data resource for research on the epidemiology of disabilities.

However, caution is needed when using the KNDRS due to the following limitations. As the KNDRS is based on a disability measurement method (e.g., a medical report, such as an impairment table [31]), it may be limited in other aspects of disability, such as loss of function and social relationships. According to a study by Okoro et al. [32], 61 million adults in the United States live with a disability, corresponding to 26% of the total population. Korea has a significantly lower proportion of registered people with disabilities (less than 5% of the total population) than the United States, which is thought to be due to the strict medical criteria used for registration in the national disability registration system. Nevertheless, this approach has the advantage of leveraging medical and statistical data. In addition, the presence of objective medical criteria for disability registration in Korea enables more objective definitions and assessment criteria of disability severity by conducting medical and public health research on people with disabilities.

## CONCLUSION

The KNDRS was established in 1989 to provide social welfare benefits based on predefined criteria for disability registration and objective medical assessment via a disability grading system. This system was criticized for being limited and not comprehensive for disabilities according to the current ICF definition of disability and underwent major reforms in 2019. The accuracy of its disability information is enabled by predefined criteria and a strict medical assessment and registration process. Moreover, it is connected to a national healthcare database, making it a promising resource for healthcare research on people with disabilities.

## Figures and Tables

**Figure f4-epih-45-e2023053:**
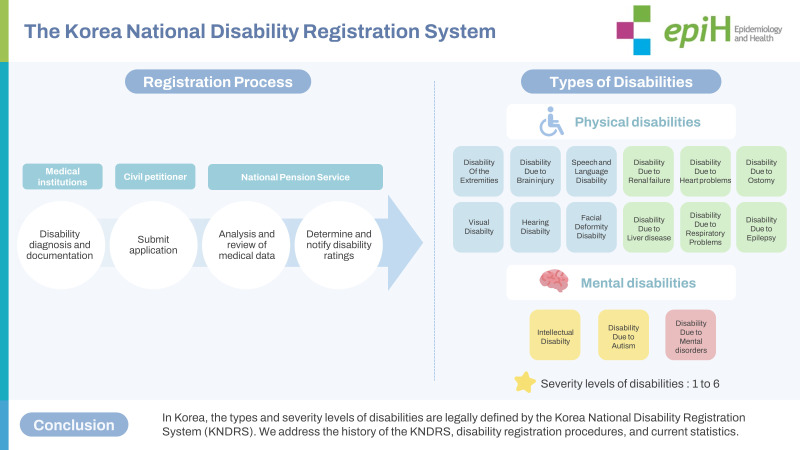


**Table 1.** Korean classification of disabilities

Parts	Components	Chapters	Categories
Physical disabilities	Impairments of external body function	Disability of the extremities	Amputation, joint disorders, dysfunction of extremities, spine disorders, and other disorders such as deformities
		Disability due to brain injury	Multiple disabilities due to brain injury
		Visual disability	Vision impairment, visual field defect
		Hearing disability	Hearing impairment, balance disorder
		Speech and language disability	Speech disability, language disability
		Facial deformity disability	Disability due to facial deformation such as scarring, depression, and/or thickening
	Impairments of internal body function	Disability due to renal failure	On dialysis or after kidney transplantation
		Disability due to heart problems	Marked limitation of physical activity due to heart dysfunction
		Disability due to liver disease	Marked limitation of physical activity due to chronic or severe liver dysfunction
		Disability due to respiratory problems	Marked limitation of physical activity due to chronic or severe pulmonary dysfunction
		Disability due to ostomy	Marked limitation of physical activity due to stoma
		Disability due to epilepsy	Marked limitation of physical activity due to chronic or severe epilepsy
Mental disabilities	Developmental impairments	Intellectual disability	IQ below 70
		Disability due to autism	Autism, childhood disintegrative disorders, etc.
	Psychiatric impairments	Disability due to mental disorders	Schizophrenia, schizoaffective disorder, bipolar affective disorder, recurrent depressive disorder, etc.

IQ, intelligence quotient.

**Table 2.** Medical institutions and specialty physicians for diagnosing disabilities

Types of disability	Medical institutions and specialty physicians
Disability of the extremities	- Amputation: physicians at a medical institution with X-ray facilities
	- Other extremity disorders: rehabilitation medicine physician, orthopedic surgeon, neurosurgeon, neurologist, or rheumatologist at a medical institution with diagnostic equipment such as X-ray facilities
Disability due to brain injury	Rehabilitation medicine physician, neurosurgeon, or neurologist
Visual disability	Ophthalmologist at a medical institution that can measure visual acuity and visual field defects
Hearing disability	Otorhinolaryngologist at a medical institution with audiometric booths
Speech and language disability	- Rehabilitation medicine physician
	- Otorhinolaryngologist, psychiatrist, or neurologist at a medical institution with speech–language pathologist
	- Maxillofacial surgeon
Facial deformity disability	Plastic surgeon, dermatologist, general surgeon (burn specialty), or maxillofacial surgeon
Disability due to renal failure	- Physician at a medical institution attended for dialysis for more than 3 mo prior to the diagnosis of disability
	- Physician at a medical institution attended for dialysis for more than 1 mo prior to the diagnosis of disability in the absence of such a doctor mentioned above (dialysis records of at least 3 mo should be checked)
	- Internal medicine physician or general surgeon (transplant) in cases of kidney transplant
Disability due to heart problems	Cardiologist, pediatrician, or thoracic surgeon who has provided treatment for at least 1 yr prior to the diagnosis of disability
Disability due to liver disease	Hepatologist, general surgeon (hepatobiliary), or pediatrician who has provided treatment for at least 2 mo prior to the diagnosis of disability
Disability due to respiratory problems	Pulmonologist, allergologist, thoracic surgeon, pediatrician, or occupational medicine specialist who has provided treatment for at least 2 mo prior to the diagnosis of disability
Disability due to ostomy	General surgeon, gynecologist, urologist, or internal medicine physician
Disability due to epilepsy	Neurologist, neurosurgeon, psychiatrist, pediatrician, or pediatric neurologist who has provided treatment for at least 6 mo prior to the diagnosis of disability
Intellectual disabilities	Psychiatrist, neurologist, or rehabilitation medicine physician
Disability due to autism	Child and adolescent psychiatrist
Disability due to mental disorders	- Psychiatrist who has provided regular treatment for at least 1 yr prior to the diagnosis of disability
	- Psychiatrist who has provided regular treatment for at least 3 mo prior to the diagnosis of disability in the absence of such a doctor mentioned above (mental health records for at least 1 yr should be checked)

**Table 3.** Number of registered disabled people by sex and type of disability in 2021

Type of disability	Total	Male	Female
Total	2,644,700	1,528,280	1,116,420
Disability of the extremities	1,191,462	691,136	500,326
Disability due to brain injury	248,308	142,600	105,708
Visual disability	251,620	149,321	102,299
Hearing disability	411,749	216,505	195,244
Speech and language disability	23,064	16,374	6,690
Facial deformity disability	2,712	1,582	1,130
Disability due to renal failure	102,135	60,527	41,608
Disability due to heart problems	5,166	3,340	1,826
Disability due to liver disease	14,433	10,119	4,314
Disability due to respiratory problems	11,541	8,503	3,038
Disability due to ostomy	16,012	9,856	6,156
Disability due to epilepsy	7,077	3,866	3,211
Intellectual disability	221,557	133,375	88,182
Disability due to autism	33,650	28,218	5,432
Disability due to mental disorders	104,214	52,958	51,256

## SUPPLEMENTARY MATERIALS

Supplementary Material 1. Gradual expansion of disability types

Supplementary Material 2 Definitions of severity degree in upper extremity amputation

Supplementary Material 3 Definitions of severity degree in lower extremity amputation

Supplementary Material 4 Definitions of severity degree in upper extremity joint disorders

Supplementary Material 5 Definitions of severity degree in lower extremity joint disorders

Supplementary Material 6 Definitions of severity degree in dysfunction of upper extremities

Supplementary Material 7 Definitions of severity degree in dysfunction of lower extremities

Supplementary Material 8 Definitions of severity degree in spine disorders

Supplementary Material 9 Definitions of severity degree in other disorders such as deformities

Supplementary Material 10 Modified Barthel Index

Supplementary Material 11 Definitions of severity degree in disabilities due to brain injury

Supplementary Material 12 Definitions of severity degree in visual disability

Supplementary Material 13 Definitions of severity degree in hearing disability

Supplementary Material 14 Definitions of severity degree in balance disorders

Supplementary Material 15 Definitions of severity degree in speech and language disability

Supplementary Material 16 Definitions of severity degree in facial deformity disability

Supplementary Material 17 Definitions of severity degree in disability due to renal failure

Supplementary Material 18 Definitions of severity degree in disability due to heart problems

Supplementary Material 19 Definitions of severity degree in disability due to liver disease

Supplementary Material 20 Definitions of severity degree in disability due to respiratory problems

Supplementary Material 21 Definitions of severity degree in disability due to ostomy

Supplementary Material 22 Definitions of severity degree in disability due to epilepsy in adults

Supplementary Material 23 Definitions of severity degree in disability due to epilepsy of children

Supplementary Material 24 Definitions of severity degree in intellectual disability

Supplementary Material 25 Definitions of severity degree in disability due to autism

Supplementary Material 26 Definitions of severity degree in disability due to mental disorder

Supplementary Material 27 The proportion of disability type by region in South Korea
