# Supplementary material for: The Korea National Disability Registration System
Source: Epidemiol Health. 2023 May 11;45:e2023053. doi: 10.4178/epih.e2023053 (PMC10482564; doi:10.4178/epih.e2023053)
Supplement: Supplementary Material 1. — Gradual expansion of disability types [file epih-45-e2023053-Supplementary-1.docx]

**Supplementary** **Material 1.** Gradual expansion of disability types

| 1989 | Limited to extremity disability, visual disability, hearing disability, speech and language disability, and intellectual disability. (5 categories) |
| --- | --- |
| 2000 | In addition to the above items, disability due to brain injury, autism, mental disorder, renal failure, or heart problems were added. (10 categories) |
| 2003 | In addition to the above items, facial deformity disability and disability due to liver disease, respiratory problems, ostomy, or epilepsy were added. (15 categories) |
