# Supplementary material for: The Korea National Disability Registration System
Source: Epidemiol Health. 2023 May 11;45:e2023053. doi: 10.4178/epih.e2023053 (PMC10482564; doi:10.4178/epih.e2023053)
Supplement: Supplementary Material 2 — Definitions of severity degree in upper extremity amputation [file epih-45-e2023053-Supplementary-2.docx]

**Supplementary Material 2.** Definitions of severity degree in upper extremity amputation

| Grade | | Definitions |
| --- | --- | --- |
| Level | Number |  |
| 1 | 1 | Amputation above the wrist joint of both arms |
| 2 | 1 | Amputation of both thumbs above the IP joint and all 2^nd^ to 5^th^ fingers above the PIP joint |
|  | 2 | Amputation above the elbow joint of one arm |
| 3 | 1 | Amputation of both thumbs above the IP joint and the 2^nd^ finger above the PIP joint |
|  | 2 | Amputation of one thumb above the IP joint and all 2^nd^ to 5^th^ fingers above the PIP joint |
| 4 | 1 | Amputation of both thumbs above the IP joint |
|  | 2 | Amputation of one thumb above the IP joint and the 2^nd^ finger above the PIP joint |
|  | 3 | Amputation of one thumb above the IP joint and another two fingers above the PIP joint |
| 5 | 1 | Amputation of one thumb above the IP joint and another finger above the PIP joint |
|  | 2 | Amputation of one thumb above the MCP joint |
|  | 3 | Amputation of three fingers including the 2^nd^ finger above the PIP joint |
| 6 | 1 | Amputation of one thumb above the IP joint |
|  | 2 | Amputation of two fingers including the 2^nd^ finger above the PIP joint |
|  | 3 | Amputation of all 3^rd^ to 5^th^ fingers of one hand above the PIP joint |

IP, Interphalangeal; PIP, proximal interphalangeal; MCP, metacarpophalangeal
