# Supplementary material for: The Korea National Disability Registration System
Source: Epidemiol Health. 2023 May 11;45:e2023053. doi: 10.4178/epih.e2023053 (PMC10482564; doi:10.4178/epih.e2023053)
Supplement: Supplementary Material 3 — Definitions of severity degree in lower extremity amputation [file epih-45-e2023053-Supplementary-3.docx]

**Supplementary Material 3.** Definitions of severity degree in lower extremity amputation

| Grade | | Definitions |
| --- | --- | --- |
| Level | Number |  |
| 1 | 2 | Amputation of both legs above the knee joint |
| 2 | 3 | Amputation of both legs above the ankle joint |
| 3 | 3 | Amputation of both legs above the transverse tarsal joint (Chopart joint) |
|  | 4 | Amputation of one leg above the knee joint |
| 4 | 4 | Amputation of both legs above the tarsometatarsal joint (Lisfranc joint) |
|  | 5 | Amputation of one leg above the ankle joint |
| 5 | 4 | Amputation of both big toes above the interphalangeal joint and the 2^nd^ to 5^th^ toes above the proximal interphalangeal joint |
|  | 5 | Amputation of one leg above the transverse tarsal joint (Chopart joint) |
| 6 | 4 | Amputation of one leg above the tarsometatarsal joint (Lisfranc joint) |
