# Supplementary material for: The Korea National Disability Registration System
Source: Epidemiol Health. 2023 May 11;45:e2023053. doi: 10.4178/epih.e2023053 (PMC10482564; doi:10.4178/epih.e2023053)
Supplement: Supplementary Material 4 — Definitions of severity degree in upper extremity joint disorders [file epih-45-e2023053-Supplementary-4.docx]

**Supplementary Material 4.** Definitions of severity degree in upper extremity joint disorders

| Grade | | Definitions |
| --- | --- | --- |
| Level | Number |  |
| 1 | 1 | ROM of all three major joints of both arms decreased by ≥75% |
| 2 | 1 | ROM of all three major joints of one arm decreased by ≥75% |
|  | 2 | ROM of two of three major joints of each arm decreased by ≥75% |
|  |  | ROM of all three major joints of both arms decreased by ≥50% and <75% |
|  | 3 | ROM of all fingers of both hands decreased by ≥75% |
| 3 | 1 | ROM of two of three major joints of each arm decreased by ≥50% and <75% |
|  |  | ROM of all three major joints of both arms decreased by ≥25% and <50% |
|  | 2 | ROM of the thumbs and 2^nd^ fingers of both hands decreased by ≥75% |
|  | 3 | ROM of all fingers of one hand decreased by ≥75% |
|  | 4 | ROM of two of three major joints of one arm decreased by ≥75% |
|  |  | ROM of all three major joints of one arm decreased by ≥50% and <75% |
| 4 | 1 | ROM of one of three major joints of one arm decreased by ≥75% |
|  |  | ROM of thumbs of both hands decreased by ≥75% |
|  | 2 | ROM of thumb and 2^nd^ finger of one hand decreased by ≥75% |
|  | 3 | ROM of three fingers of one hand including thumb or 2^nd^ finger decreased by ≥75% |
|  | 4 | ROM of four fingers of one hand including thumb or 2^nd^ finger decreased by ≥50% and <75% |
| 5 | 1 | ROM of two of three major joints of one arm decreased by ≥50% and <75% |
|  |  | ROM of all three major joints of one arm decreased by ≥25% and <50% |
|  | 2 | ROM of thumbs of both hands decreased by ≥50% and <75% |
|  | 3 | ROM of thumb of one hand decreased by ≥75% |
|  | 4 | ROM of thumb and 2^nd^ finger of one hand decreased by ≥50% and <75% |
|  | 5 | ROM of three fingers of one hand including thumb or 2^nd^ finger decreased by ≥50% and <75% |
| 6 | 1 | ROM of one of three major joints of one arm decreased by ≥50% and <75% |
|  |  | ROM of thumb of one hand decreased by ≥50% and <75% |
|  | 2 | ROM of two fingers of one hand including 2^nd^ finger decreased by ≥75% |
|  | 3 | ROM of two fingers of one hand including thumb decreased by ≥50% and <75% |
|  | 4 | ROM of 3^rd^, 4^th^, and 5^th^ fingers of one hand decreased by ≥75% |

ROM, range of motion

The three major joints refer to the shoulder, elbow, and wrist.
