# Supplementary material for: The Korea National Disability Registration System
Source: Epidemiol Health. 2023 May 11;45:e2023053. doi: 10.4178/epih.e2023053 (PMC10482564; doi:10.4178/epih.e2023053)
Supplement: Supplementary Material 5 — Definitions of severity degree in lower extremity joint disorders [file epih-45-e2023053-Supplementary-5.docx]

**Supplementary Material 5.** Definitions of severity degree in lower extremity joint disorders

| Grade | | Definitions |
| --- | --- | --- |
| Level | Number |  |
| 1 | 2 | ROM of all three major joints of both legs decreased by ≥75% |
| 2 | 4 | ROM of two of three major joints of each leg decreased by ≥75% |
|  |  | ROM of all three major joints of both legs decreased by ≥50% and <75% |
| 3 | 5 | ROM of all three major joints of one leg decreased by ≥75% |
| 4 | 1 | ROM of two of three major joints of each leg decreased by ≥50% and <75% |
|  |  | ROM of all three major joints of both legs decreased by ≥25% and <50% |
|  | 2 | ROM of the hip or knee joint of one leg decreased by ≥90% |
|  | 5 | ROM of two of three major joints of one leg decreased by ≥75% |
|  |  | ROM of all three major joints of one leg decreased by ≥50% and <75% |
| 5 | 1 | ROM of the hip or knee joint of one leg decreased by ≥75% and <90% |
|  | 2 | ROM of the ankle joint of one leg ≥90% |
|  | 6 | ROM of two of three major joints of one leg decreased by ≥50% and <75% |
|  |  | ROM of all three major joints of one leg decreased by ≥25% and <50% |
|  | 7 | ROM of all toes of both feet decreased by ≥75% |
| 6 | 2 | ROM of the hip or knee joint of one leg decreased by ≥50% and <75% |
|  | 3 | ROM of the ankle joint of one leg decreased by ≥75% and <90% |

ROM, range of motion

The three major joints refer to the hip, knee, and ankle.
