# Supplementary material for: The Korea National Disability Registration System
Source: Epidemiol Health. 2023 May 11;45:e2023053. doi: 10.4178/epih.e2023053 (PMC10482564; doi:10.4178/epih.e2023053)
Supplement: Supplementary Material 6 — Definitions of severity degree in dysfunction of upper extremities [file epih-45-e2023053-Supplementary-6.docx]

**Supplementary Material 6.** Definitions of severity degree in dysfunction of upper extremities

| Grade | | Definitions |
| --- | --- | --- |
| Level | Number |  |
| 1 | 1 | Complete paralysis of both arms (manual muscle strength grade^*^ 0, 1) |
| 2 | 1 | Complete paralysis of one arm (grade 0, 1) |
|  | 2 | Minimal movement with both nearly paralyzed arms (grade 2) |
|  | 3 | Complete paralysis of all fingers of both hands (grade 0, 1) |
| 3 | 1 | Paralysis of both arms, which are moderately mobile but not functional (grade 3) |
|  | 2 | Complete paralysis of the thumb and 2^nd^ fingers of both hands (grade 0, 1) |
|  | 3 | Complete paralysis of all fingers of one hand (grade 0, 1) |
|  | 4 | Minimal movement with one paralyzed arm (grade 2) |
| 4 | 1 | Complete paralysis of the thumbs of both hands (grade 0, 1) |
|  | 2 | Complete paralysis of the thumb and 2^nd^ finger of one hand (grade 0, 1) |
|  | 3 | Complete paralysis of three fingers of one hand, including the thumb or 2^nd^ finger (grade 0, 1) |
|  | 4 | Paralysis of four fingers of one hand including the thumb or 2^nd^ finger, which are moderately mobile but not functional (grade 3) |
| 5 | 1 | Paralysis of one arm, which is moderately mobile but not functional (grade 3) |
|  | 2 | Paralysis of the thumbs of both hands, which are moderately mobile but not functional (grade 3) |
|  | 3 | Complete paralysis of the thumb of one hand (grade 0, 1) |
|  | 4 | Paralysis of the thumb and 2^nd^ finger of one hand, which are moderately mobile but not functional (grade 3) |
|  | 5 | Paralysis of three fingers of one hand including the thumb or 2^nd^ finger, which are moderately mobile but not functional (grad 3) |
| 6 | 1 | Paralysis of thumb of one hand, which is moderately mobile but not functional (grade 3) |
|  | 2 | Complete paralysis of two fingers of one hand, including the 2^nd^ finger (grade 0, 1) |
|  | 3 | Paralysis of two fingers of one hand including the thumb, which are moderately mobile but not functional (grade 3) |
|  | 4 | Complete paralysis of 3^rd^, 4^th^, and 5^th^ fingers of one hand (grade 0, 1) |

^*^ Manual muscle strength grade: 5 (normal), complete range of motion against gravity with full resistance; 4 (good), complete range of motion against gravity with some resistance; 3 (fair), complete range of motion against gravity with no resistance; 2 (poor), complete range of motion with gravity eliminated; 1 (trace), evidence of slight contractility with no evidence of joint motion even with gravity eliminated; 0 (zero), no evidence of muscle contractility
