# Supplementary material for: The Korea National Disability Registration System
Source: Epidemiol Health. 2023 May 11;45:e2023053. doi: 10.4178/epih.e2023053 (PMC10482564; doi:10.4178/epih.e2023053)
Supplement: Supplementary Material 7 — Definitions of severity degree in dysfunction of lower extremities [file epih-45-e2023053-Supplementary-7.docx]

**Supplementary Material 7.** Definitions of severity degree in dysfunction of lower extremities

| Grade | | Definitions |
| --- | --- | --- |
| Level | Number |  |
| 1 | 2 | Complete paralysis of both legs (manual muscle strength grade^*^ 0, 1) |
| 2 | 4 | Minimal movement with both nearly paralyzed legs (grade 2) |
| 3 | 5 | Complete paralysis of one leg (grade 0, 1) |
| 4 | 1 | Paralysis of both legs, which are moderately mobile but not functional (grade 3) |
|  | 5 | Slight movement with one nearly paralyzed leg (grade 2) |
| 5 | 6 | Paralysis of one leg, which is moderately mobile but not functional (grade 3) |
| 5 | 7 | Complete paralysis of all toes of both feet (grade 0, 1) |

^*^Manual muscle strength grade: 5 (normal), complete range of motion against gravity with full resistance; 4 (good), complete range of motion against gravity with some resistance; 3 (fair), complete range of motion against gravity with no resistance; 2 (poor), complete range of motion with gravity eliminated; 1 (trace), evidence of slight contractility with no evidence of joint motion even with gravity eliminated; 0 (zero), no evidence of muscle contractility
