# Supplementary material for: The Korea National Disability Registration System
Source: Epidemiol Health. 2023 May 11;45:e2023053. doi: 10.4178/epih.e2023053 (PMC10482564; doi:10.4178/epih.e2023053)
Supplement: Supplementary Material 8 — Definitions of severity degree in spine disorders [file epih-45-e2023053-Supplementary-8.docx]

**Supplementary Material 8.** Definitions of severity degree in spine disorders

| Grade | | Definitions |
| --- | --- | --- |
| Level | Number |  |
| 2 | 5 | ROM of the C and T-L spines decreased by ≥4/5 of normal |
|  | 6 | Complete stiffness of the C and T-L spines due to AS |
| 3 | 1 | ROM of the C or T-L spine decreased by ≥4/5 of normal |
| 4 | 1 | ROM of the C or T-L spine decreased by ≥3/5 of normal |
| 5 | 8 | ROM of the C or T-L spine decreased by ≥2/5 of normal |
|  | 9 | Complete stiffness of the C and T or T and L spines due to AS |
| 6 | 5 | ROM of the C or T-L spine decreased by ≥1/5 of normal |
|  | 6 | Complete stiffness of the C or L spine due to AS |

ROM, range of motion; C, cervical; T-L, thoracolumbar; T, thoracic; L, lumbar; AS, ankylosing spondylitis
