# Supplementary material for: The Korea National Disability Registration System
Source: Epidemiol Health. 2023 May 11;45:e2023053. doi: 10.4178/epih.e2023053 (PMC10482564; doi:10.4178/epih.e2023053)
Supplement: Supplementary Material 9 — Definitions of severity degree in other disorders such as deformities [file epih-45-e2023053-Supplementary-9.docx]

**Supplementary Material 9.** Definitions of severity degree in other disorders such as deformities

| Grade | | Definitions |
| --- | --- | --- |
| Level | Number |  |
| 5 | 1 | Leg-length discrepancy ≥10 cm or 1/10 of the unaffected leg |
| 6 | 1 | Leg-length discrepancy ≥ 5 cm or 1/15 of the unaffected leg |
|  | 2 | Scoliosis with ≥40 degrees of curvature |
|  | 3 | Kyphosis with ≥60 degrees of curvature |
|  | 4 | A male aged ≥18 years and <145 cm in height, no longer growing |
|  | 5 | A female aged ≥18 years and <140 cm in height, no longer growing |
|  | 6 | Symptoms of dwarfism due to achondroplasia are evident^*^ |

^*^Only applicable to subjects aged ≥2 years
