# Supplementary material for: The Korea National Disability Registration System
Source: Epidemiol Health. 2023 May 11;45:e2023053. doi: 10.4178/epih.e2023053 (PMC10482564; doi:10.4178/epih.e2023053)
Supplement: Supplementary Material 10 — Modified Barthel Index [file epih-45-e2023053-Supplementary-10.docx]

**Supplementary Material 10.** Modified Barthel Index

| Items | Code | | | | |
| --- | --- | --- | --- | --- | --- |
|  | Unable to perform task | Fully dependent | Moderate help required | Minimal help required | Fully independent |
| Personal hygiene^*^ | 0 | 1 | 3 | 4 | 5 |
| Bathing self | 0 | 1 | 3 | 4 | 5 |
| Feeding | 0 | 2 | 5 | 8 | 10 |
| Toilet | 0 | 2 | 5 | 8 | 10 |
| Stair climbing | 0 | 2 | 5 | 8 | 10 |
| Dressing^**^ | 0 | 2 | 5 | 8 | 10 |
| Bowel control | 0 | 2 | 5 | 8 | 10 |
| Bladder control | 0 | 2 | 5 | 8 | 10 |
| Chair/bed transfer^†^ | 0 | 3 | 8 | 12 | 15 |
| Ambulation | 0 | 3 | 8 | 12 | 15 |
| Wheelchair | 0 | 1 | 3 | 4 | 5 |

^*^Personal hygiene includes washing, combing hair, brushing teeth, shaving, etc.

^**^Dressing includes button locking and unlocking, wearing a belt, tying and untying shoelaces, etc.

^†^Chair/bed transfer includes moving from bed to chair or from chair to bed or sitting in bed, etc.
