# Supplementary material for: The Korea National Disability Registration System
Source: Epidemiol Health. 2023 May 11;45:e2023053. doi: 10.4178/epih.e2023053 (PMC10482564; doi:10.4178/epih.e2023053)
Supplement: Supplementary Material 11 — Definitions of severity degree in disabilities due to brain injury [file epih-45-e2023053-Supplementary-11.docx]

**Supplementary Material 11.** Definitions of severity degree in disabilities due to brain injury

| Grade | Definitions |
| --- | --- |
| 1 | Unable to walk independently and requires total assistance from others |
|  | Unable to perform any ordinary activities due to complete paralysis of both arms and requires total assistance from others |
|  | Unable to perform any ordinary activities due to complete paralysis of one arm and one leg and requires total assistance from others |
|  | Modified Barthel Index is ≤32 points and requires total assistance from others to perform all ordinary activities including ambulation |
| 2 | Unable to perform any ordinary activities due to complete paralysis of one arm and requires total assistance from others |
|  | Unable to use all fingers of both hands due to complete paralysis and joint contracture and requires total assistance from others |
|  | Modified Barthel Index of 33-53 points and requires assistance from others to perform most ordinary activities including ambulation |
| 3 | Unable to use all fingers of one hand due to complete paralysis and joint contracture and requires total assistance from others |
|  | Unable to walk due to complete paralysis of one leg and requires much assistance from others |
|  | Modified Barthel Index of 54-69 points and is unable to perform ordinary activities independently and requires partial assistance from others |
| 4 | Modified Barthel Index of 70-80 points and is able to perform ordinary activities but occasionally requires assistance from others |
| 5 | Modified Barthel Index of 81-89 points and is able to perform most ordinary activities independently but sometimes requires assistance from others |
| 6 | Modified Barthel Index of 81-89 points and is able to perform most ordinary activities independently but sometimes requires more time |
