# Supplementary material for: The Korea National Disability Registration System
Source: Epidemiol Health. 2023 May 11;45:e2023053. doi: 10.4178/epih.e2023053 (PMC10482564; doi:10.4178/epih.e2023053)
Supplement: Supplementary Material 12 — Definitions of severity degree in visual disability [file epih-45-e2023053-Supplementary-12.docx]

**Supplementary Material 12.** Definitions of severity degree in visual disability

| Grade | | Definitions |
| --- | --- | --- |
| Level | Number |  |
| 1 | 1 | Visual acuity of better eye ≤20/1000 |
| 2 | 1 | Visual acuity of better eye ≤20/500 |
| 3 | 1 | Visual acuity of better eye ≤20/300 |
|  | 2 | Visual field of each eye ≤5 degrees in any direction |
| 4 | 1 | Visual acuity of better eye ≤20/200 |
|  | 2 | Visual field of each eye ≤10 degrees in any direction |
| 5 | 1 | Visual acuity of better eye ≤ 20/100 |
|  | 2 | Sum of visual field of both eyes ≤50% of normal |
| 6 | N/A | Visual acuity of worse eye ≤20/1000 |

N/A, not applicable
