# Supplementary material for: The Korea National Disability Registration System
Source: Epidemiol Health. 2023 May 11;45:e2023053. doi: 10.4178/epih.e2023053 (PMC10482564; doi:10.4178/epih.e2023053)
Supplement: Supplementary Material 13 — Definitions of severity degree in hearing disability [file epih-45-e2023053-Supplementary-13.docx]

**Supplementary Material 13.** Definitions of severity degree in hearing disability

| Grade | | Definitions |
| --- | --- | --- |
| Level | Number |  |
| 2 | N/A | Hearing loss^*^ of both ears ≥90 dB |
| 3 | N/A | Hearing loss^*^ of both ears ≥80 dB |
| 4 | 1 | Hearing loss^*^ of both ears ≥70 dB |
|  | 2 | Maximum speech intelligibility ≤50% |
| 5 | N/A | Hearing loss^*^ of both ears ≥60 dB |
| 6 | N/A | Hearing loss^*^ of one ear ≥80 dB and that of the other ear ≥40 dB |

^*^Average of four frequencies (0.5, 1, 2, and 4 kHz) on pure tone audiometry. N/A, not applicable
