# Supplementary material for: The Korea National Disability Registration System
Source: Epidemiol Health. 2023 May 11;45:e2023053. doi: 10.4178/epih.e2023053 (PMC10482564; doi:10.4178/epih.e2023053)
Supplement: Supplementary Material 14 — Definitions of severity degree in balance disorders [file epih-45-e2023053-Supplementary-14.docx]

**Supplementary Material 14.** Definitions of severity degree in balance disorders

| Grade | Definitions |
| --- | --- |
| 3 | Loss of bilateral vestibular function  and unable to stand up with both eyes open or collapses while walking 10 m in a straight line with both eyes open  and unable to perform any ordinary activity other than self-care without assistance from others |
| 4 | Decrease in bilateral vestibular function  and stopping halfway to balance while walking 10 m in a straight line with both eyes open  and only able to take care of oneself, walk, and perform simple activities |
| 5 | Decrease in bilateral or unilateral vestibular function  and moving more than 60 cm from center while walking 10 meters in a straight line with both eyes open  and unable to perform complex activities |

If clinically unavoidable, diagnosis of balance disorders can be made by walking 6 m.
