# Supplementary material for: The Korea National Disability Registration System
Source: Epidemiol Health. 2023 May 11;45:e2023053. doi: 10.4178/epih.e2023053 (PMC10482564; doi:10.4178/epih.e2023053)
Supplement: Supplementary Material 15 — Definitions of severity degree in speech and language disability [file epih-45-e2023053-Supplementary-15.docx]

**Supplementary Material 15.** Definitions of severity degree in speech and language disability

| Grade | | Definitions |
| --- | --- | --- |
| Level | Number |  |
| 3 | 1 | Unable to produce sounds  or conduct a simple conversation using an electrolarynx or esophageal voice |
|  | 2 | Stuttering that interrupts ≥97% of the flow of speech |
|  | 3 | Articulation disorder with consonant accuracy <30% |
|  | 4 | Expressive language quotient <25 with minimal or no meaningful speech^*^ |
|  | 5 | Receptive language quotient <25 with minimal or no understating of simple words^*^ |
| 4 | 1 | Speech disorder with partially possible vocalization (pitch, intensity, and quality) |
|  | 2 | Stuttering that interrupts the flow of speech (children: 41–96%, adults: 24–96%) |
|  | 3 | Articulation disorder with a consonant accuracy of 30–75% |
|  | 4 | Expressive language quotient of 25–65 with highly limited expression^*^ |
|  | 5 | Receptive language quotient of 25–65 with notably minimal understanding^*^ |

^*^Any language or speech disorder caused by intellectual disability or autism is excluded.
