# Supplementary material for: The Korea National Disability Registration System
Source: Epidemiol Health. 2023 May 11;45:e2023053. doi: 10.4178/epih.e2023053 (PMC10482564; doi:10.4178/epih.e2023053)
Supplement: Supplementary Material 16 — Definitions of severity degree in facial deformity disability [file epih-45-e2023053-Supplementary-16.docx]

**Supplementary Material 16.** Definitions of severity degree in facial deformity disability

| Grade | | Definitions |
| --- | --- | --- |
| Level | Number |  |
| 2 | 1 | Deformation of ≥90% of the facial area |
|  | 2 | Deformation of ≥60% of the facial area and loss of ≥2/3 of the nose shape |
| 3 | 1 | Deformation of ≥75% of the facial area |
|  | 2 | Deformation of ≥50% of the facial area and loss of ≥2/3 of the nose shape |
| 4 | 1 | Deformation of ≥60% of the facial area |
|  | 2 | Loss of ≥2/3 of the nose shape |
|  | 3 | Deformation of ≥45% of the facial area and loss of ≥1/3 of the nose shape |
| 5 | 1 | Deformation of ≥45% of the facial area |
|  | 2 | Loss of ≥1/3 of the nose shape |
