# Supplementary material for: The Korea National Disability Registration System
Source: Epidemiol Health. 2023 May 11;45:e2023053. doi: 10.4178/epih.e2023053 (PMC10482564; doi:10.4178/epih.e2023053)
Supplement: Supplementary Material 17 — Definitions of severity degree in disability due to renal failure [file epih-45-e2023053-Supplementary-17.docx]

**Supplementary Material 17.** Definitions of severity degree in disability due to renal failure

| Grade | Definitions |
| --- | --- |
| 2 | On hemodialysis or peritoneal dialysis due to chronic kidney disease for ≥3 months |
| 5 | Kidney transplantation |
