# Supplementary material for: The Korea National Disability Registration System
Source: Epidemiol Health. 2023 May 11;45:e2023053. doi: 10.4178/epih.e2023053 (PMC10482564; doi:10.4178/epih.e2023053)
Supplement: Supplementary Material 18 — Definitions of severity degree in disability due to heart problems [file epih-45-e2023053-Supplementary-18.docx]

**Supplementary Material 18.** Definitions of severity degree in disability due to heart problems

| Grade | Definitions |
| --- | --- |
| 1 | Angina syndrome may be present at rest with persistently reduced heart function  Sum of the clinical findings and test results^*^ ≥30 points |
| 2 | Angina syndrome causes marked limitation in ordinary physical activities with persistently reduced heart function  Sum of the clinical findings and test results^*^ 25–29 points |
| 3 | Angina syndrome causes slight limitation in ordinary activities with persistently reduced heart function  Sum of the clinical findings and test results^*^ 20–24 points |
| 4 | Heart transplantation |

It is limited to those who have been diagnosed with heart disease for more than one year.

^*^ Clinical findings and test results

1) Treadmill test (7 METS (metabolic equivalents) or more: 1 point, 5–7 METS: 2 points, 2.5–5 METS: 4 points, 2.5 METS or less: 5 points)

2) Classification of angina severity (No angina with ordinary physical activities; however, strenuous activity may cause symptoms: 1 point, Angina causes a slight limitation on ordinary physical activities: 2 points, Angina causes marked limitation on ordinary physical activities: 4 points, Angina occurs with any physical activity and may be present at rest: 5 points)

3) Ejection fraction of left ventricle (41–50%: 1 point, 31–40%: 3 points, 21–30%: 5 points, 20% or less: 8 points

4) Chest x-ray (out of 5 points), electrocardiogram (out of 5 points) and cyanosis score (out of 3 points)

5) Personal history of heart surgery or intervention (out of 8 points)

6) Hospitalization history (out of 8 points)

7) Treatment history within the last 9 months (out of 3 points)
