# Supplementary material for: The Korea National Disability Registration System
Source: Epidemiol Health. 2023 May 11;45:e2023053. doi: 10.4178/epih.e2023053 (PMC10482564; doi:10.4178/epih.e2023053)
Supplement: Supplementary Material 19 — Definitions of severity degree in disability due to liver disease [file epih-45-e2023053-Supplementary-19.docx]

**Supplementary Material 19.** Definitions of severity degree in disability due to liver disease

| Grade | | Definitions |
| --- | --- | --- |
| Level | Number |  |
| 1 | N/A | Residual liver function classified as Child–Pugh C class^*^ due to chronic liver disease (liver cirrhosis or hepatocellular carcinoma, etc.)  and with hepatic encephalopathy or diuretic refractory ascites |
| 2 | N/A | Residual liver function classified as Child-Pugh C class due to chronic liver disease (liver cirrhosis or hepatocellular carcinoma, etc.)  and with history of hepatic encephalopathy or of spontaneous bacterial peritonitis within the last 2 years |
| 3 | 1 | Residual liver function classified as Child–Pugh C class due to chronic liver disease (liver cirrhosis or hepatocellular carcinoma, etc.) |
|  | 2 | Residual liver function classified as Child–Pugh B class due to chronic liver disease (liver cirrhosis or hepatocellular carcinoma, etc.)  and with hepatic encephalopathy or diuretic refractory ascites |
| 5 | N/A | Liver transplantation |

N/A, not applicable

^*^ Child–Pugh class estimates degree of cirrhosis considering serum bilirubin, serum albumin, ascites, neurological abnormalities, and prothrombin time. (5–6 points: class A, 7–9 points: class B, ≥10 points: class C)
