# Supplementary material for: The Korea National Disability Registration System
Source: Epidemiol Health. 2023 May 11;45:e2023053. doi: 10.4178/epih.e2023053 (PMC10482564; doi:10.4178/epih.e2023053)
Supplement: Supplementary Material 20 — Definitions of severity degree in disability due to respiratory problems [file epih-45-e2023053-Supplementary-20.docx]

**Supplementary Material 20.** Definitions of severity degree in disability due to respiratory problems

| Grade | | Definitions |
| --- | --- | --- |
| Level | Number |  |
| 1 | 1 | With chronic respiratory failure, oxygen therapy is required even at rest  and ^*^FEV1 ≤25% predicted  or arterial oxygen saturation at rest less than 55 mmHg (without oxygen supply) |
|  | 2 | Requires ventilator all day with tracheostomy tube due to chronic respiratory disease |
| 2 | N/A | Too breathless to leave the house and breathless when dressing or undressing  and FEV1 ≤30% predicted  or arterial oxygen saturation at rest ≤60 mmHg (without oxygen supply) |
| 3 | N/A | Short of breath after walking a few minutes on level surface  and FEV1 ≤40% predicted  or arterial oxygen saturation at rest ≤65 mmHg (without oxygen supply) |
| 5 | 1 | Lung transplantation |
|  | 2 | Pleural fistula |

N/A, not applicable

^*^FEV1 = forced expiratory volume in one second
