# Supplementary material for: The Korea National Disability Registration System
Source: Epidemiol Health. 2023 May 11;45:e2023053. doi: 10.4178/epih.e2023053 (PMC10482564; doi:10.4178/epih.e2023053)
Supplement: Supplementary Material 21 — Definitions of severity degree in disability due to ostomy [file epih-45-e2023053-Supplementary-21.docx]

**Supplementary Material 21.** Definitions of severity degree in disability due to ostomy

| Grade | | Definitions |
| --- | --- | --- |
| Level | Number |  |
| 2 | 1 | Colostomy with urostomy or vesicostomy  and with complications in more than one of the stomas such as enterocutaneous fistula or urinary dysfunction |
|  | 2 | Colostomy or urostomy with enterocutaneous fistula and urinary dysfunction |
|  | 3 | Ileostomy for defecation |
| 3 | 1 | Colostomy with urostomy or vesicostomy |
|  | 2 | Colostomy or urostomy with enterocutaneous fistula or urinary dysfunction |
| 4 | 1 | Colostomy or urostomy |
|  | 2 | Vesicostomy with enterocutaneous fistula |
| 5 | N/A | Vesicostomy |

N/A, not applicable
