# Supplementary material for: The Korea National Disability Registration System
Source: Epidemiol Health. 2023 May 11;45:e2023053. doi: 10.4178/epih.e2023053 (PMC10482564; doi:10.4178/epih.e2023053)
Supplement: Supplementary Material 22 — Definitions of severity degree in disability due to epilepsy in adults [file epih-45-e2023053-Supplementary-22.docx]

**Supplementary Material 22.** Definitions of severity degree in disability due to epilepsy in adults

| Grade | Definitions |
| --- | --- |
| 2 | ≥8 severe seizures^*^ in one month, occurring ≥6 times per year despite active treatment  and seizures require serious medical care for a respiratory disorder, aspiration pneumonia, severe exhaustion, headache, nausea, and/or cognitive impairment  and totally dependent on others in daily and social life |
| 3 | ≥5 severe seizures in one month (≥10 mild seizures^**^), occurring ≥6 times per year despite active treatment  and seizures require medical care for a respiratory disorder, aspiration pneumonia, severe exhaustion, headache, nausea, and/or cognitive impairment  and dependent on extensive assistance of others in daily and social life |
| 4 | More than one severe seizure in one month (or ≥2 mild seizures), occurring ≥6 times per year despite active treatment  and marked limitations in interpersonal relationships |
| 5 | ≥1 severe seizure per month (or ≥2 mild seizures), occurring ≥3 times per year despite active treatment  and mild limitation in interpersonal relationships |

^*^Severe seizures include 1) generalized tonic-clonic or tonic or clonic seizures, 2) seizures with loss of balance, 3) seizures with loss of consciousness lasting more than 3 min, 4) seizures causing an accident or trauma

^**^Mild seizures are not severe seizures and are subject to the disability grade system
