# Supplementary material for: The Korea National Disability Registration System
Source: Epidemiol Health. 2023 May 11;45:e2023053. doi: 10.4178/epih.e2023053 (PMC10482564; doi:10.4178/epih.e2023053)
Supplement: Supplementary Material 23 — Definitions of severity degree in disability due to epilepsy of children [file epih-45-e2023053-Supplementary-23.docx]

**Supplementary Material 23.** Definitions of severity degree in disability due to epilepsy of children

| Grade | | Definitions |
| --- | --- | --- |
| Level | Number |  |
| 2 | 1 | ≥8 generalized seizures per month |
|  | 2 | ≥4 seizures per month causing head drop or a fall |
|  | 3 | ≥4 seizures per month with epileptic encephalopathy such as infantile spasm or Lennox–Gastaut syndrome |
|  | 4 | ≥4 myoclonic seizures per month causing a fall |
| 3 | 1 | 4–7 generalized seizures per month |
|  | 2 | 1–3 seizures per month causing head drop or a fall |
|  | 3 | 1–3 seizures per month with epileptic encephalopathy such as infantile spasm or Lennox-Gastaut syndrome |
|  | 4 | 1–3 myoclonic seizures per month causing a fall |
|  | 5 | ≥10 focal seizures per month |
| 4 | 1 | 1–3 generalized seizures per month |
|  | 2 | 1–5 seizures in 6 months causing head drop or a fall |
|  | 3 | 1–5 seizures in 6 months with epileptic encephalopathy such as infantile spasm or Lennox–Gastaut syndrome |
|  | 4 | 1–5 myoclonic seizures in 6 months causing a fall |
|  | 5 | 1–9 focal seizures per month |

Absence seizures are excluded from the disability grade system.

Mild myoclonic seizures are excluded from the disability grade system; only severe myoclonic seizures causing a fall are included.
