# Supplementary material for: The Korea National Disability Registration System
Source: Epidemiol Health. 2023 May 11;45:e2023053. doi: 10.4178/epih.e2023053 (PMC10482564; doi:10.4178/epih.e2023053)
Supplement: Supplementary Material 24 — Definitions of severity degree in intellectual disability [file epih-45-e2023053-Supplementary-24.docx]

**Supplementary Material 24.** Definitions of severity degree in intellectual disability

| Grade | Definitions |
| --- | --- |
| 1 | IQ<35 and has marked difficulty in adapting to daily life and social life, relying on the assistance of others |
| 2 | IQ 35–50 and can learn simple activities and hold a job with the supervision and help of others |
| 3 | IQ 50–70 and capable of social and vocational rehabilitation through education |

IQ, Intelligence quotient
