# Supplementary material for: The Korea National Disability Registration System
Source: Epidemiol Health. 2023 May 11;45:e2023053. doi: 10.4178/epih.e2023053 (PMC10482564; doi:10.4178/epih.e2023053)
Supplement: Supplementary Material 25 — Definitions of severity degree in disability due to autism [file epih-45-e2023053-Supplementary-25.docx]

**Supplementary Material 25.** Definitions of severity degree in disability due to autism

| Grade | Definitions |
| --- | --- |
| 1 | Pervasive developmental disorders (autism spectrum disorders) by ICD-10 criteria  and IQ ≤70  and GAS score ≤20 |
| 2 | Pervasive developmental disorders (autism spectrum disorders) by ICD-10 criteria  and IQ ≤70  and GAS score 21–40 |
| 3 | Pervasive developmental disorders (autism spectrum disorders) by ICD-10 criteria  and IQ ≥71  and GAS score 41–50 |

IQ, Intelligence quotient

The GAS (goal assessment scale) measures daily functioning of children with autism spectrum disorder.
