# Supplementary material for: The Korea National Disability Registration System
Source: Epidemiol Health. 2023 May 11;45:e2023053. doi: 10.4178/epih.e2023053 (PMC10482564; doi:10.4178/epih.e2023053)
Supplement: Supplementary Material 26 — Definitions of severity degree in disability due to mental disorder [file epih-45-e2023053-Supplementary-26.docx]

**Supplementary Material 26.** Definitions of severity degree in disability due to mental disorder

| Grade | | Definitions |
| --- | --- | --- |
| Level | Number |  |
| 1 | 1 | Schizophrenia with severe positive symptoms (delusion, hallucination, disorganized thinking, and bizarre behavior) and negative symptoms (social withdrawal and apathy) and marked personality changes  and totally dependent on the assistance of others in ≥3 of the criteria for disability  and GAF score ≤40 |
|  | 2 | Bipolar affective disorder with persistent or recurrent severe manic episodes (excitement, hyperactivity, disinhibition, etc.)  and totally dependent on the assistance of others in ≥3 of the criteria for disability^*^  and GAF score ≤40 |
|  | 3 | Recurrent depressive disorder with psychotic features and persistent or recurrent severe depressive episodes (negative emotions, suicidal thoughts, etc.)  and totally dependent on the assistance of others in ≥3 of the criteria for disability^*^  and GAF score ≤40 |
|  | 4 | Schizoaffective disorder satisfying criteria of 1-1 or 1-3 above |
| 2 | 1 | Schizophrenia with positive symptoms (delusion, hallucination, disorganized thinking, and bizarre behavior) and negative symptoms (social withdrawal and apathy) and moderate personality changes  and dependent on extensive assistance of others in ≥3 of the criteria for disability^*^  and GAF score 41–50 |
|  | 2 | Bipolar affective disorder with persistent or recurrent manic episodes (excitement, hyperactivity and disinhibition, etc.)  and dependent on extensive assistance of others in ≥3 of the criteria for disability^*^  and GAF score 41–50 |
|  | 3 | Recurrent depressive disorder with psychotic features and persistent or recurrent depressive episodes (negative emotions, suicidal thoughts, etc.)  and dependent on extensive assistance of others in ≥3 of the criteria for disability^*^  and GAF score 41–50 |
|  | 4 | Schizoaffective disorder satisfying criteria of 2-1 or 2-3 above |
| 3 | 1 | Schizophrenia with positive symptoms (delusion, hallucination, disorganized thinking, and bizarre behavior) and mild personality changes  and dependent on limited assistance of others in ≥3 of the criteria for disability^*^  and GAF score 51–60 |
|  | 2 | Bipolar affective disorder with persistent or recurrent mild manic episodes (excitement, hyperactivity and disinhibition, etc.)  and dependent on limited assistance of others in ≥3 of the criteria for disability^*^  and GAF score 51–60 |
|  | 3 | Recurrent depressive disorder with persistent or recurrent depressive episodes (negative emotions, suicidal thoughts, etc.)  and dependent on limited assistance of others in ≥3 of the criteria for disability^*^  and GAF score 51-60 |
|  | 4 | Schizoaffective disorder satisfying criteria of 3-1 or 3-3 above |

Limited to those who have been diagnosed with psychiatric disorders for more than one year.

^*^ The criteria for disability (1. Proper feeding 2. Keeping oneself clean such as by toileting, bathing, and washing. 3. Appropriate communication skills and cooperative interpersonal relationships. 4. Taking medication at the correct dose and time by oneself. 5. Managing financial matters. 6. Using transportation)

The GAF (global assessment of functioning) is designed to measure the degree to which a person's symptoms affect their daily life.
