# Supplementary material for: The Korea National Disability Registration System
Source: Epidemiol Health. 2023 May 11;45:e2023053. doi: 10.4178/epih.e2023053 (PMC10482564; doi:10.4178/epih.e2023053)
Supplement: Supplementary Material 27 — The proportion of disability type by region in South Korea [file epih-45-e2023053-Supplementary-27.docx]

**Supplementary Material 27.** The proportion of disability type by region in South Korea

|  | Seoul | Busan | Daegu | Incheon | Gwang-ju | Dae-jeon | Ulsan | Sejong | Gyeon-ggi | Gang-won | Chung-  buk | Chung-  nam | Jeon-buk | Jeon-nam | Gyeong-buk | Gyeong-nam | Jeju |
| --- | --- | --- | --- | --- | --- | --- | --- | --- | --- | --- | --- | --- | --- | --- | --- | --- | --- |
| **Types of disability** |  |  |  |  |  |  |  |  |  |  |  |  |  |  |  |  |  |
| Extremities disability | 46.0 | 46.7 | 43.6 | 49.5 | 44.5 | 46.3 | 47.5 | 49.0 | 49.1 | 50.7 | 47.9 | 48.5 | 50.3 | 50.6 | 46.5 | 50.1 | 40.9 |
| Visual disability | 10.7 | 10.4 | 9.8 | 9.7 | 10.5 | 9.7 | 9.7 | 9.9 | 9.7 | 9.4 | 9.3 | 9.1 | 8.8 | 9.9 | 9.2 | 9.2 | 11.8 |
| Hearing disability | 12.8 | 11.8 | 15.8 | 13.4 | 13.9 | 13.0 | 14.4 | 12.9 | 11.7 | 13.4 | 13.3 | 15.3 | 13.1 | 13.8 | 16.4 | 12.3 | 16.4 |
| Language disability | 0.8 | 0.8 | 0.8 | 0.9 | 0.8 | 0.8 | 0.8 | 0.7 | 0.8 | 0.7 | 0.7 | 0.8 | 0.9 | 0.7 | 0.7 | 0.7 | 1.1 |
| Intellectual disability | 6.7 | 6.6 | 8.0 | 7.1 | 9.6 | 8.6 | 8.2 | 8.6 | 7.8 | 8.2 | 10.5 | 8.8 | 9.2 | 8.3 | 9.0 | 8.1 | 9.0 |
| Due to brain injury | 10.6 | 11.6 | 10.6 | 9.3 | 9.8 | 10.3 | 9.7 | 8.7 | 10.0 | 9.2 | 9.0 | 8.3 | 9.2 | 8.1 | 9.1 | 9.6 | 9.9 |
| Due to autism | 1.5 | 1.0 | 0.9 | 1.0 | 1.1 | 1.3 | 1.1 | 1.3 | 1.3 | 0.7 | 0.7 | 0.7 | 0.5 | 0.5 | 0.6 | 0.9 | 1.5 |
| Due to mental disorder | 4.2 | 4.7 | 4.9 | 3.3 | 4.4 | 4.3 | 2.8 | 3.7 | 3.5 | 3.1 | 3.8 | 4.0 | 4.2 | 4.0 | 4.2 | 4.2 | 3.6 |
| Due to renal failure | 4.2 | 4.0 | 3.6 | 3.7 | 3.2 | 3.5 | 3.6 | 3.0 | 3.8 | 2.7 | 2.9 | 2.5 | 2.2 | 2.3 | 2.6 | 3.1 | 3.6 |
| Due to heart problems | 0.3 | 0.3 | 0.2 | 0.2 | 0.2 | 0.2 | 0.4 | 0.2 | 0.2 | 0.1 | 0.2 | 0.2 | 0.1 | 0.1 | 0.2 | 0.1 | 0.2 |
| Due to respiratory problems | 0.5 | 0.6 | 0.4 | 0.5 | 0.4 | 0.4 | 0.5 | 0.4 | 0.5 | 0.6 | 0.4 | 0.4 | 0.3 | 0.4 | 0.4 | 0.4 | 0.7 |
| Due to liver disease | 0.6 | 0.4 | 0.5 | 0.4 | 0.5 | 0.4 | 0.6 | 0.5 | 0.6 | 0.4 | 0.4 | 0.4 | 0.3 | 0.4 | 0.5 | 0.4 | 0.5 |
| Facial deformity disability | 0.1 | 0.1 | 0.1 | 0.1 | 0.1 | 0.1 | 0.1 | 0.1 | 0.1 | 0.1 | 0.1 | 0.1 | 0.1 | 0.1 | 0.1 | 0.1 | 0.1 |
| Due to ostomy | 0.7 | 0.6 | 0.5 | 0.6 | 0.5 | 0.5 | 0.4 | 0.6 | 0.6 | 0.6 | 0.6 | 0.6 | 0.5 | 0.5 | 0.5 | 0.5 | 0.5 |
| Due to epilepsy | 0.3 | 0.3 | 0.3 | 0.3 | 0.4 | 0.4 | 0.2 | 0.2 | 0.3 | 0.2 | 0.3 | 0.2 | 0.3 | 0.3 | 0.2 | 0.2 | 0.3 |
